# Controlling Nutritional Status (CONUT) score for predicting all-cause mortality in patients who underwent percutaneous coronary intervention after acute myocardial infarction: a cohort study

**DOI:** 10.3389/fnut.2025.1604470

**Published:** 2025-07-08

**Authors:** Yang Song, Su Han, Shiru Zhang, Yalun Yuan, Meizhu Wang, Zhaoqing Sun

**Affiliations:** Department of Cardiology, Shengjing Hospital of China Medical University, Shenyang, Liaoning, China

**Keywords:** malnutrition, CONUT, acute myocardial infarction, percutaneous coronary intervention, prognosis

## Abstract

**Background:**

Controlling Nutritional Status (CONUT) score, a novel marker reflecting the malnutrition, has been demonstrated to predict all-cause mortality and major adverse cardiovascular events (MACE) in a wide range of diseases. The research intends to assess the clinical effects of malnutrition on patients who have percutaneous coronary intervention (PCI) after acute myocardial infarction (AMI).

**Methods:**

In this retrospective observational study, we consecutively enrolled 3258 patients diagnosed with AMI from 2010 to 2016. Patients were categorized into three groups based on the CONUT score: normal, mild malnutrition, and moderate and severe malnutrition. The primary outcome was all-cause mortality. We develop cox proportional hazards models to investigate the relationship between the CONUT score and all-cause mortality among patients who underwent PCI after AMI.

**Results:**

According to the assessment via the CONUT score, a total of 43.7% patients experienced mild malnutrition, and 4.8% patients experienced moderate and severe malnutrition. During a median follow-up period of 8.6 years, there were 610 patients (18.7%) suffered from all-cause mortality. As malnutrition severity intensified, the occurrence of the primary endpoint saw a steady rise. After adjusting for multiple variables, the group classified with moderate and severe malnutrition exhibited an odds ratio of 1.56 (95% CI 1.13 to 2.15, *p* = 0.007) for the primary endpoint. Incorporating the CONUT score augments the prognostic accuracy of the GRACE risk score in predicting all-cause mortality (Absolute Integrated Discrimination Improvement = 0.008, *p* < 0.001; Category-free Net Reclassification Improvement = 0.144, *p* = 0.001).

**Conclusion:**

Malnutrition is prevalent among patients with AMI and is significantly associated with an increased incidence of all-cause mortality. As a nutritional assessment tool, the CONUT score effectively aids in risk stratification and predicts poor prognosis in patients. Additional prospective clinical trials are required to evaluate the influence of nutritional interventions on outcomes in patients undergoing PCI after AMI.

## 1 Introduction

Acute myocardial infarction (AMI) is a major contributor to death globally and imposes significant costs related to medical treatment and work-related disabilities (1). Even though percutaneous coronary intervention (PCI) represents a sophisticated advanced therapy for AMI, this disease remains a prevalent and deadly form of cardiovascular illness, continuing to pose significant challenges to public health (2, 3). To enhance outcomes for AMI patients following PCI, it is crucial to further recognize those at heightened risk and strive to minimize the occurrence of negative events.

Malnutrition has been consistently identified by various studies as an independent risk factor for cardiovascular diseases, involving coronary artery disease (CAD), heart failure, cardiomyopathy, non-valvular atrial fibrillation (AF) and peripheral vascular disease (4–9). It is significantly associated with elevated all-cause mortality and increased incidence of major adverse cardiovascular events (MACE). Previous studies have suggested that the elevated mortality risk of AMI associated with malnutrition may be attributable to inflammatory mechanisms (10, 11). Inflammation can impair endothelial cell function, increase lipoprotein permeability, and facilitate the progression of atherosclerosis. Besides, inflammation may further exacerbate malnutrition (12, 13). Stenvinkel et al. reported that inflammation and malnutrition are closely linked to arteriosclerosis, and they have referenced Malnutrition-Inflammation-Atherosclerosis (MIA) syndrome (14). Currently, the American Heart Association (AHA) has demonstrated through the implementation of nutritional intervention measures that such approaches can reduce high-sensitivity C-reactive protein (CRP) level, thereby effectively improving patient prognosis (15). Consequently, timely and accurate assessment of patients’ nutritional status holds critical importance in clinical practice. With the aid of multiple nutritional assessment tools, the malnutrition status of patients can not only be quantified but also serve as an auxiliary factor for risk stratification. It provides clinicians with a more efficient approach to address patients’ malnutrition status. The Controlling Nutritional Status (CONUT) score is a nutritional assessment tool which comprises serum albumin, total cholesterol and lymphocyte counts, and its simplicity and effectiveness have been acknowledged (16–18). Nonetheless, the research surrounding the CONUT score, which serves to forecast the outcomes for patients experiencing AMI and undergoing PCI, remains sparse.

Accordingly, we intend to illustrate the incidence and long-term outcomes related to malnutrition in AMI patients following PCI while utilizing the CONUT score. Furthermore, the study aims to explore if the CONUT score can enhance the prognostic capabilities of the GRACE risk score concerning all-cause mortality.

## 2 Materials and methods

### 2.1 Study population

This retrospective observational study involved the inclusion of 3398 patients who were admitted to the cardiovascular department of ShengJing Hospital, part of China Medical University, and were ultimately underwent PCI after diagnosing with AMI during the period from January 2010 to December 2016. Patients who lost to follow-up (*n* = 29), lacked crucial baseline laboratory data (*n* = 75) were excluded. We also excluded 8 patients with a diagnosis of malignancy, 6 patients with a diagnosis of infection (19), and 22 patients with a diagnosis of severe liver dysfunction. Ultimately, 3258 patients were screened for analysis (Supplementary Figure 1). AMI was defined as acute myocardial injury with clinical evidence of acute myocardial ischemia according to Fourth Universal Definition of Myocardial Infarction by European Society of Cardiology (20). The study has been approved by Research Ethics Committee of ShengJing Hospital of China Medical University and conducted according to the principles of the Declaration of Helsinki.

### 2.2 Nutritional screening tools and clinical measurements

After enrollments, this study utilizes the CONUT points-scoring system to categorize the population into three groups. The CONUT scoring system is designed to evaluate nutritional health, incorporating three critical indicators: serum albumin, total cholesterol and lymphocyte cells (17, 18). Three laboratory variables were converted to the corresponding scores and add the scores together can obtain the CONUT scores. A score ranging from 0 to 1 was defined as normal nutritional status, while scores ranging from 2 to 4, 5 to 8, and 9 to 12 respectively indicate mild, moderate, and severe malnutrition (Supplementary Table 1).

The Laboratory data were measured by laboratory clinic in ShengJing Hospital of China Medical University. Fasting venous blood samples were gathered from all patients within 24 h of admission. Lymphocytes and hemoglobin were conducted using an automated blood counter (Unicell DxH800 Coulter, Beckman Coulter Corp., United States) with optical light scatter counting. The total cholesterol, low-density lipoprotein cholesterol (LDL-C), high-density lipoprotein cholesterol (HDL-C), serum albumin and creatine were calculated from an enhanced immunonephelometric assay on an automated analyzer (AU5800, Beckman Coulter Corp., United States). The left ventricular ejection fraction was measured using standardized echocardiography performed by the Department of Ultrasound at ShengJing Hospital, China Medical University within the first 48 h of admission (21).

### 2.3 Outcomes and follow-up

The primary outcome was all-cause mortality. We conducted follow-up via telephone interviews and review of medical records. The terminal survival status within the cohort was determined in October 2023. The follow-up phase concluded on 31 October 2023, or upon the occurrence of death.

### 2.4 Statistical analysis

Continuous variables which is normally distributed were expressed as mean ± SD, while those without normally distributed variables were expressed as a median with interquartile range (IQR). Categorical variables were expressed as *n* (%). Continuous variables compared between groups using the ANOVA and the Kruskal-Wallis test. Categorical variables were compared using the χ2 test. Time-to-event outcomes were illustrated using Kaplan-Meier survival curves, and the log-rank test was employed to evaluate differences in survival between groups. Univariable and two multivariate cox regression models were performed to assess the independent associations of CONUT with all-cause mortality. Model 1 of multivariate cox regression was adjusted for age and sex, while Model 2 was adjusted for age, sex, hypertension, diabetes, heart failure, prior AF, type of AMI (ST and non-ST segment elevation myocardial infarction), previous history of PCI, previous history of coronary-artery-bypass-grafting (CABG), left main trunk (LM)/multivessel coronary artery disease, calcification, chronic total occlusion (CTO), LDL-C, HDL-C, creatine, left ventricular ejection fraction (LVEF), dual antiplatelet therapy (DAPT), statin, angiotensin converting enzyme inhibitor (ACEI) and beta-blocker. Results were represented as hazard ratios (HRs) with associated 95% confidence intervals (CIs). In addition, we stratified the study population by age, gender, type of MI, and the presence or absence of comorbidities such as hypertension, diabetes, heart failure, and AF, and conducted multivariate COX regression analysis for each subgroup. In order to compare whether CONUT scores have the capacity to enhance the prognostic value in all-cause mortality in contrast to the traditional GRACE risk score, we utilized Harrell C-statistics, category-free net reclassification improvement (NRI) and absolute integrated discrimination improvement (IDI) to access the predictable value. All *p-values* were two-sided, and *p-values* < 0.05 were regarded as statistically significant. Above statistical analysis was conducted using IBM SPSS Statistics 26.

## 3 Results

### 3.1 Baseline characteristics

Table 1 outlines the baseline characteristics of the study cohort. From the total of 3258 patients included, 1737 had STEMI (53.3%), and 1522 had NSTEMI (46.7%). Most of them were male (70.9%), and the median age was 62.0 ± 11.8 years. Nearly 50% of the patients reported being smokers, and over one-third exhibited left main trunk or multivessel coronary artery disease. Among these patients, 48.5% (*n* = 1580) of patients were identified as malnourished using the CONUT scoring system. In addition, 43.7% (*n* = 1,424) had mild malnutrition and 4.8% (*n* = 156) had moderate and severe malnutrition, respectively. Patients with a lower nutritional status tended to be older, and had diabetes mellitus, heart failure, a diagnosis of STEMI and worse Killip class. In laboratory data, the moderate and severe malnutrition group exhibited lower levels of lymphocytes, total cholesterol (TC), albumin, and hemoglobin. The moderate and severe malnutrition group also exhibited a higher GRACE risk score, suggesting a heightened likelihood of all-cause mortality. The usage of medications following the onset of AMI showed no significant variation among the three different groups.

**TABLE 1:** Baseline characteristics of study population.

Characteristics	Total (*n* = 3258)	Normal (*n* = 1678)	Mild (*n* = 1424)	Moderate and severe (*n* = 156)	*P*-value
Age, years	62.0 ± 11.8	60.5 ± 11.8	63.2 ± 11.6	66.3 ± 12.2	<0.001
Male, *n* (%)	2312 (70.9%)	1154 (68,8%)	1044 (73.3%)	113 (72.4%)	0.016
**Comorbidities, *n* (%)**					
Hypertension	1847 (56.7%)	946 (56.4%)	811 (57.0%)	89 (57.1%)	0.949
Diabetes	1061 (32.6%)	512 (30.5%)	492 (34.6%)	57 (36.5%)	0.040
Heart failure	164 (5.0%)	56 (3.3%)	88 (6.2%)	18 (11.5%)	<0.001
Prior AF	116 (3.6%)	49 (2.9%)	59 (4.1%)	7 (4.5%)	0.410
Smokers	1711 (52.5%)	887 (52.9%)	751 (52.7%)	74 (47.4%)	0.343
**Type of MI, *n* (%)**					
NSTEMI	1522 (46.7%)	768 (45.8%)	687 (48.2%)	66 (42.3%)	0.190
STEMI	1737 (53.3%)	910 (54.2%)	737 (51.8%)	90 (57.7%)	
Killip ≥ 2 class, *n* (%)	265 (8.1%)	120 (7.1%)	121 (8.5%)	24 (15.4%)	0.001
**Laboratory data**					
Lymphocyte, × 10∧9/L	1.93 ± 0.81	2.25 ± 0.70	1.62 ± 0.79	1.34 ± 0.79	<0.001
TC, mg/dL	175.7 ± 43.7	192.7 ± 36.5	159.8 ± 41.9	137.4 ± 50.8	<0.001
Albumin, g/L	38.8 ± 3.8	40.0 ± 3.0	38.0 ± 3.7	32.7 ± 4.5	<0.001
HDL-C, mg/dL	38.9 ± 10.7	40.4 ± 10.9	37.5 ± 10.2	35.0 ± 9.9	<0.001
LDL-C, mg/dL	112.4 ± 36.8	125.0 ± 32.8	100.3 ± 34.2	88.3 ± 48.9	<0.001
Creatine, mg/dL (25th, 75th)	0.8 (0.7–1.0)	0.8 (0.7–1.0)	0.9 (0.7–1.0)	0.9 (0.7–1.2)	0.001
Hemoglobin, g/L	135.8 ± 17.7	138.8 ± 16.5	133.8 ± 18.0	123.5 ± 18.8	<0.001
LVEF (%)	56.3 ± 9.5	57.0 ± 8.8	55.7 ± 10.0	54.4 ± 11.0	<0.001
**Lesion type, *n* (%)**					
LM/multivessel	1106 (33.9%)	547 (32.6%)	506 (35.5%)	52 (33.3%)	0.234
Calcification	203 (6.7%)	87 (5.6%)	107 (8.1%)	8 (5.5%)	0.019
CTO	363 (18.4%)	166 (16.8%)	179 (20.5%)	19 (17.6%)	0.111
Previous PCI, *n* (%)	201 (6.2%)	80 (4.8%)	110 (7.7%)	10 (6.4%)	0.003
Previous CABG, *n* (%)	12 (0.4%)	5 (0.3%)	6 (0.4%)	1 (0.6%)	0.721
**Medications, *n* (%)**					
DAPT	3160 (97.0%)	1624 (96.8%)	1373 (96.4%)	151 (96.8%)	0.254
ACEI/ARB	1900 (58.3%)	975 (58.1%)	831 (58.4%)	93 (59.6%)	0.936
Statin	3144 (96.5%)	1632 (96.7%)	1368 (96.1%)	164 (96.5%)	0.546
Beta-blocker	1757 (53.9%)	918 (54.7%)	762 (53.5%)	77 (49.4%)	0.326
GRACE risk score	142.5 ± 34.4	138.7 ± 33.2	145.7 ± 34.9	153.8 ± 36.4	<0.001

Data are presented as median (IQR), or number (%), or median with interquartile range (25th to 75th percentiles). AF, atrial fibrillation; MI, myocardial infarction, STEMI, ST-elevated myocardial infarction; NSTEMI, non-ST-elevated myocardial infarction; PCI, percutaneous coronary intervention; CABG, coronary-artery-bypass-grafting; LM, left main trunk; CTO, chronic total occlusion; TC, total cholesterol; HDL-C, high-density lipoprotein cholesterol; LDL-C, low-density lipoprotein cholesterol; LVEF, left ventricular ejection fraction; DAPT, dual antiplatelet therapy; ACEI, angiotensin converting enzyme inhibitor; ARB, angiotensin II receptor blocker.

### 3.2 Prognostic factors for patients with different risks of malnutrition and outcomes

Following the adjustment for variables such as age and sex, model 1 indicated that patients within the moderate and severe malnutrition category faced a heightened risk relative to those classified as adequately nourished (HR 1.66, 95% CI 1.23 to 2.25, *p* = 0.001). Furthermore, after adjusting for all variables outlined in Supplementary Table 2, model 2 indicated that patients classified within the moderate and severe malnutrition category exhibited an elevated risk in comparison to those in the normal nutrition category (HR 1.56, 95% CI 1.13 to 2.15, *p* = 0.007) (Table 2).

**TABLE 2:** Cox proportional hazards models of CONUT to forecast all-cause mortality.

CONUT, as a categories variable	Univariate analysis			Multivariate analysis model 1			Multivariate analysis model 2		
	*HR*	*95%CI*	*P-value*	*HR*	*95%CI*	*P-value*	*HR*	*95%CI*	*P-value*
Nourished	Reference			Reference			Reference		
Mild risk	1.34	1.13–1.58	0.001	1.18	1.01–1.40	0.049	1.12	0.94–1.34	0.208
Moderate and severe risk	2.29	1.70–3.09	<0.001	1.66	1.23–2.25	0.001	1.56	1.13–2.15	0.007

Model 1 adjusted for age, sex; Model 2 adjusted for age, sex, hypertension, diabetes, heart failure, prior AF, history of smoking, type of acute myocardial infarction (ST and non-ST segment elevation myocardial infarction), previous PCI, previous CABG, LM/multivessel coronary artery disease, calcification, CTO, LDL-C, HDL-C, creatine, LVEF, DAPT, statin, ACEI, beta-blocker; CONUT, controlling nutritional status; AF, atrial fibrillation; PCI, percutaneous coronary intervention; CABG, coronary-artery-bypass-grafting; LM, left main trunk; CTO, chronic total occlusion; HDL-C, high-density lipoprotein cholesterol; LDL-C, low-density lipoprotein cholesterol; LVEF, left ventricular ejection fraction; DAPT, dual antiplatelet therapy; ACEI, angiotensin converting enzyme inhibitor.

**FIGURE 1:**
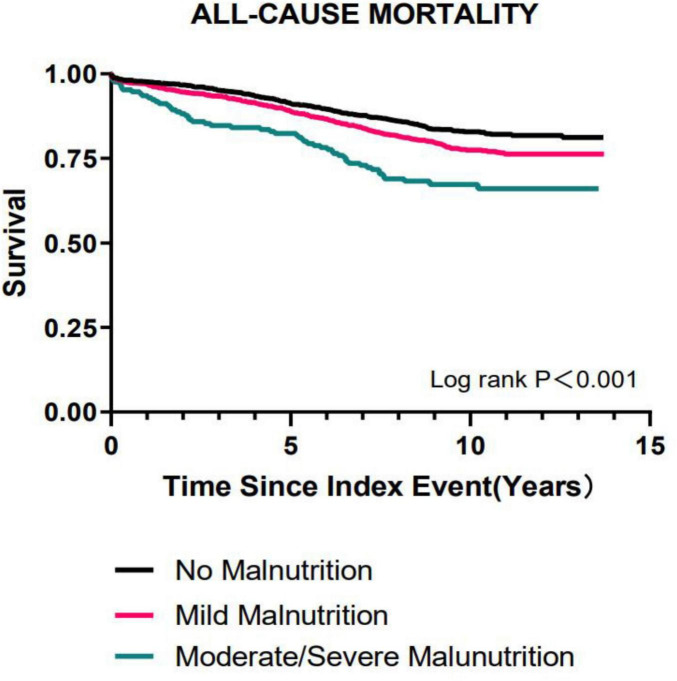
Analysis of the Kaplan-Meier curves for all-cause mortality categorized by the CONUT score among patients who underwent PCI after AMI.

**FIGURE 2:**
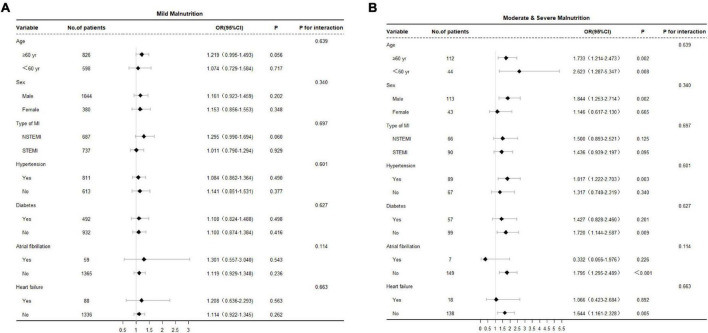
**(A)** Stratified analysis of the mild malnutrition group and all-cause mortality among patients who underwent PCI after AMI. Adjusted for research center, age, sex, hypertension, diabetes, atrial fibrillation and heart failure. **(B)** Stratified analysis of the moderate and severe malnutrition group and all-cause mortality among patients who underwent PCI after AMI. Adjusted for research center, age, sex, hypertension, diabetes, atrial fibrillation and heart failure.

Moreover, during a median follow-up of 8.6 years, 610 patients died after discharge. On Kaplan-Meier curve analysis, an increased all-cause mortality was observed among malnourished patients compared to nourished patients in the long-term prognosis. The moderate and severe malnutrition group exhibited the highest mortality (Figure 1). In subgroup analysis, the results showed that malnutrition had no significant difference in the prognosis impact on different characteristic populations (Figures 2A, B).

**FIGURE 3:**
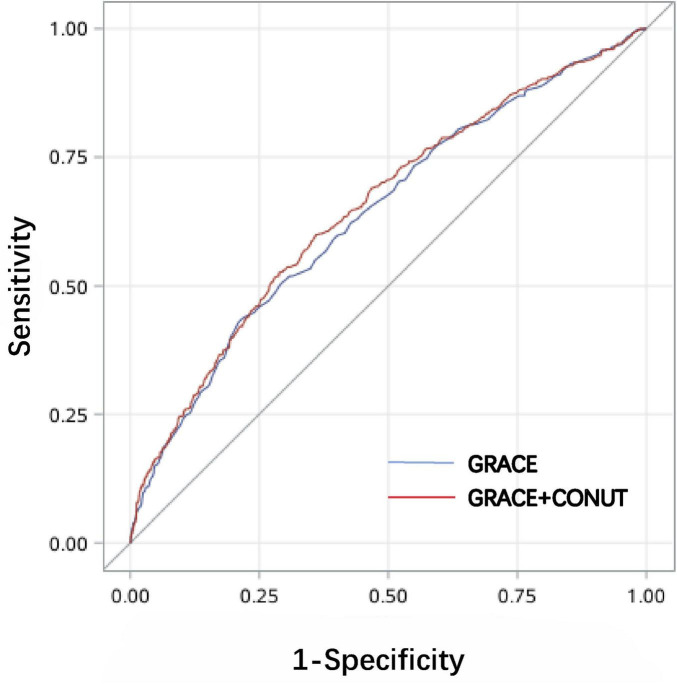
ROC curve analysis for both the GRACE risk score and the combined CONUT + GRACE risk score.

Furthermore, we calculated the corresponding areas under the curve (AUC) and constructed the receiver operating characteristic (ROC) curve for both the GRACE risk score and the combined CONUT + GRACE risk score using C-statistics. The AUCs were 0.640 (*p* < 0.001, 95% CI: 0.615 to 0.664) and 0.651 (*p* < 0.001,95% CI: 0.627 to 0.676) respectively. The new model formed by GRACE risk score combined with CONUT can improve the prediction efficiency of GRACE risk score (NRI = 0.144, *p* = 0.001; IDI = 0.008, *p* < 0.001) (Figure 3 and Table 3).

**TABLE 3:** Evaluating the effectiveness of the integrated the CONUT score alongside the GRACE risk Score for forecasting all-cause mortality.

Model	C-statistic	P-value	NRI	P-value	IDI	P-value
GRACE	0.640	Ref	Ref		Ref	
GRACE + CONUT	0.651	0.033	0.144	0.001	0.008	<0.001

## 4 Discussion

In the analysis conducted retrospectively, we observed that (1) patients suffering from AMI frequently experience malnutritional conditions, with our study revealing that nearly 48.5% of the patients experienced malnutrition, with 4.8% experienced moderate and severe malnutrition. (2) Malnutrition is an independent predictor of all-cause mortality in patients who underwent PCI after AMI, and the CONUT score, as a nutritional assessment tool, can assist in risk stratification. (3) The CONUT score can increase the prognostic value of the GRACE risk score for predicting all-cause mortality in AMI patients who underwent PCI. These aspects underscore the critical need for mitigating malnutrition risks and stress the importance of prompt intervention.

The incidence of malnutrition is very common among patients with AMI. Huang Y et al. found that malnutrition accounted for 53.8% in a cohort of 1180 AMI patients based on the PNI score (22). Basta G et al. showed that 55% of patients were malnourished in their evaluation of the nutritional status within a STEMI cohort (23). In our study, the prevalence of malnutrition was comparable to that reported in prior research. However, Wada et al. found that a significant proportion, approximately 20% of patients with coronary artery disease who underwent PCI were identified as being at high risk for malnutrition (24). Another study examined malnutrition prevalence in a group of 207 patients diagnosed with NSTEMI, uncovering a rate of 24.4% (25). The incidence of malnutrition in both studies was lower compared to our study. The potential reasons for this distinction may include different study population and different scoring system compared to our study.

Within our study cohort, it was observed that malnutrition independently contributed to all-cause mortality among patients who underwent PCI after AMI. Various previous investigations have also shown that malnutrition acts as an independent predictor of AMI when evaluated using different assessment methods. Li M et al. indicated that Prognostic Nutritional Index (PNI) was an independent predictor of long-term prognosis in elderly STEMI patients (26). Additionally, another study employed the Geriatric Nutrition Risk Index (GNRI) to gauge nutritional status, highlighting a notable connection between GNRI and unfavorable outcomes in AMI patients following PCI (27). Few studies have utilized the CONUT score to assess the relationship between malnutrition and the prognosis of patients who underwent PCI after AMI. PNI, GNRI, and CONUT score are widely recognized and commonly utilized nutritional assessment tools (28). We selected CONUT as the nutritional assessment tool for our study based on the findings of Raposeiras Roubín S et al., who evaluated the predictive value of PNI, GNRI, and CONUT for all-cause mortality and MACE in ACS patients. Their results suggested that the predictive capability of the CONUT score ranked the highest (29).

The GRACE risk score exhibits superior predictive performance for both in-hospital mortality and one-year mortality in patients with AMI (30). As AMI management and catheter devices have changed considerably since the establishment of the GRACE score, it is therefore important to develop a new scoring model for prospective risk stratification that is suitable for the current era of AMI management (31). Our research found that CONUT can improve the predictive efficiency of GRACE risk score. This may can be more effectively aid in the risk stratification of patients following PCI after AMI.

The pathophysiology of the relationship between high CONUT scores and poor clinical outcomes in AMI patients is not clearly known. The CONUT score is calculated based on the serum albumin, total cholesterol level and the lymphocyte count in the peripheral blood. The serum albumin has been confirmed by multiple studies as an indicator related to inflammation (32, 33). The activity of proinflammatory cytokines is contributed to the decrease in serum albumin levels (10, 34). On the other hand, atherosclerosis is increasingly recognized as a chronic inflammatory disease with an autoimmune component. A decrease in lymphocytes may cause immunosuppression in the patient, which can influence cardiac remodeling process after AMI and lead to poor prognosis (35–38). Meanwhile, the immune system primarily mediates its immune response by recognizing LDL antibodies, thereby generating oxidized LDL and triggering inflammation in the arterial wall (39). The above-mentioned evidence supports that the rationality of the CONUT score in assessing the prognosis of AMI patients, and indicates that there is an interaction and mutual promotion relationship between malnutrition, inflammation and immune response. All these findings strongly underscore the importance for physicians to incorporate malnutrition identification into their daily practice. Especially, patients with AMI are generally elderly, fragile patients with comorbidities. The CONUT score will provide important guidance for subsequent nutritional intervention measures. Shah B et al. indicates that dietary interventions can reduce high-sensitivity CRP level, which may have a beneficial effect on the prognosis of coronary heart disease (15). In the future, more well-designed experiments should be conducted to evaluate the specific effects of nutritional interventions on the prognosis of patients who underwent PCI after AMI.

Our study is subject to several limitations. Firstly, it was a single-center, retrospective study. All of our patients were of Chinese ethnicity, which may not reflect the prognosis observed in other ethnic groups, thereby limiting the general applicability of our findings. Secondly, we only utilized simple screening tools to assess nutritional status, without conducting direct comparisons with other well-established nutritional assessment tools, such as the Subjective Global Assessment and the Mini Nutritional Assessment. Furthermore, since the assessment of nutrition was carried out at just one point in time, it limited our ability to track the evolving nutritional conditions of the patients over the study period. Assessing long-term clinical outcomes using information collected at just one moment carries fundamental limitations. Besides, regarding medical therapy, we only conducted statistical analysis on the medication treatment of patients after discharge, without covering the medication situation before admission (such as the application of statins). This limitation may have a certain impact on the accuracy of cholesterol levels in the baseline data. Ultimately, while numerous clinically significant factors were incorporated in the multivariate evaluation, it is likely that unaccounted confounding variables still exist.

## 5 Conclusion

Malnutrition is prevalent among patients with AMI and is significantly associated with an increased incidence of all-cause mortality. The CONUT score, serving as a nutritional assessment tool, plays a key role in assisting with risk categorization and forecasting patients’ poor prognosis. The predictive capability of the GRACE risk score regarding all-cause mortality can be improved by incorporating the CONUT score. Early screening of malnutrition using the CONUT score can provide a foundation for guiding subsequent clinical nutritional interventions.

## Data Availability

The raw data supporting the conclusions of this article will be made available by the authors, without undue reservation.
