# Supplementary material for: Controlling Nutritional Status (CONUT) score for predicting all-cause mortality in patients who underwent percutaneous coronary intervention after acute myocardial infarction: a cohort study
Source: Front Nutr. 2025 Jul 8;12:1604470. doi: 10.3389/fnut.2025.1604470 (PMC12279502; doi:10.3389/fnut.2025.1604470)
Supplement: Supplementary file 1 [file Table_1.docx]

**Supplementary Figure 1. Study Flow Chart.**

**Total recruited**

(n=3398)

**Exclusion**

1. 29 cases excluded due to loss to **follow-up**

2. 75 cases **missing crucial baseline laboratory**

3. 8 patients **with a diagnosis of malignancy**

4. 6 patients **with a diagnosis of infection**

5. 22 patients **with a diagnosis of severe liver dysfunction**

**Mild**

(n=1424)

**Eligible cases included in this study**

（n=3258）

**Normal**

(n=1678）

**Moderate & Severe**

(n=156)

**Supplementary table 1. Prevalence of Malnutrition According to the CONUT Scoring System**

| **Nutritional Screening Tool** | **Risk of Malnutrition** | | | |
| --- | --- | --- | --- | --- |
|  | **Normal** | **Mild** | **Moderate** | **Severe** |
| **CONUT, points** | **0-1** | **2-4** | **5-8** | **9-12** |
| **Albumin, g/l(scores)** | **≥35.0(0)** | **30.0-34.9(2)** | **25.0-29.9(4)** | **＜25.0(6)** |
| **Lymphocyte, ×10^9/l(scores)** | **≥1.60(0)** | **1.20-1.59(1)** | **0.80-1.19(2)** | **＜0.80(3)** |
| **Total cholesterol, mmol/l(scores)** | **≥180 (0)** | **140-179(1)** | **100-139(2)** | **＜100(3)** |
| **Study Population** | **1687(51.2)** | **1437(43.6)** | **166(5.1)** | **4(0.1)** |

**Supplementary table 2. Univariable cox regression results for all-cause mortality in patients with acute myocardial infarction**

|  | *Univariable analysis* | |
| --- | --- | --- |
|  | HR (95% CI) | P value |
| Age, years | 1.06 (1.05-1.07) | ＜0.001 |
| Sex, (female vs male) | 1.49 (1.26-1.75) | ＜0.001 |
| Comorbidities |  |  |
| Hypertension | 1.26 (1.07-1.48) | 0.006 |
| Diabetes | 1.30 (1.11-1.53) | 0.001 |
| Heart failure | 2.70 (2.09-3.46) | ＜0.001 |
| Prior AF | 2.53 (1.87-3.42) | ＜0.001 |
| Smoking | 1.07(0.92-1.26) | 0.382 |
| Type of AMI |  |  |
| NSTEMI | ref | ref |
| STEMI | 1.00 (0.85-1.17) | 0.976 |
| KILLIP≥2class | 1.72 (1.35-2.19) | ＜0.001 |
| Laboratory data |  |  |
| Lymphocyte, ×10^9/L | 1.00(0.90-1.10) | 0.968 |
| Total cholesterol, mg/dl | 1.00(1.00-1.00) | 0.014 |
| Albumin, g/l | 0.91(0.89-0.93) | ＜0.001 |
| HDL-C, mg/dl | 1.01 (1.00-1.01) | 0.210 |
| LDL-C, mg/dl | 1.00 (1.00-1.01) | 0.149 |
| Creatine, μmol/l | 1.05 (1.00-1.10) | 0.063 |
| Hemoglobin, g/l | 0.98 (0.97-0.98) | ＜0.001 |
| LVEF% | 0.98 (0.97-0.99) | ＜0.001 |
| Lesion type |  |  |
| LM/multivessel | 1.57 (1.34-1.85) | ＜0.001 |
| Calcification | 1.49 (1.12-1.98) | 0.006 |
| CTO | 1.53 (1.22-1.92) | ＜0.001 |
| Previous PCI | 1.72 (1.32-2.25) | ＜0.001 |
| Previous CABG | 2.58 (1.07-6.21) | 0.035 |
| Medications |  |  |
| DAPT | 0.51(0.36-0.71) | ＜0.001 |
| ACEI/ARB | 0.95 (0.81-1.11) | 0.508 |
| Statin | 0.56 (0.40-0.78) | 0.001 |
| Beta-blocker | 0.87 (0.75-1.02) | 0.092 |
| GRACE risk score | 1.01 (1.01-1.02) | ＜0.001 |
| Malnutrition |  |  |
| Normal | Ref | Ref |
| Mild | 1.52(1.28-1.81) | ＜0.001 |
| Moderate &Severe | 2.70(2.11-3.45) | ＜0.001 |
